# Nux Vomica, a Powerful Antidote for Opium Poisoning

**Published:** 1885-12

**Authors:** A. C. Davidson

**Affiliations:** Sharon, Ga.


### NUX VOMICA—A POWERFUL ANTIDOTE FOR OPIUM POISONING.

BY A. C. DAVIDSON, M. D., SHARON, GA.

The following report has been withheld a considerable length of time in order that its value might be increased or modified by additional clinical observation.

It is hoped that the medical profession will give it at least a passing notice, seeing that so many fatal cases, accidental or otherwise, of the kind here reported are being chronicled almost daily in the public press.

Case 1.—In the month of June, 1879, I was attending in the same family the grandfather, Mr. Edmund H., aged fifty-six, who was sick with a colliquative diarrhœa consecutive to a very severe attack of cholera-morbus, and at the same time his little granddaughter, Mattie J., aged three, who was sick with a mild form of gastric remittent fever.

At my visit on the morning of the seventeenth, I prescribed, among other medicines for the grandfather, as he was accustomed to its use, several pretty large doses of the sulphate of morphia, 2 grs. each, and for little Mattie several doses of the sulphate of cinchonidia, 3 grs. each.

Inasmuch as the patients were in different rooms, entirely separated from each other, I had no idea that the medicines would become interchangeable.

The grandmother, upon whom devolved the duty of administering the medicines, by some unaccountable mistake gave the cinchonidia to the grandfather and the morphia to little Mattie. This was at 1 o'clock p. m. About 1:30 p. m. I was summoned hurriedly to see the little girl, the messenger reporting that the little patient seemed to be dying.

It being only a mile distant, I soon arrived upon the scene of confusion, for such it was, as quite a number of the neighbors had already arrived and were doing what they could to alleviate the intensely distressed condition of the family in a manner peculiar to excited people, who do not know what to do.

The grandmother met me at the front gate, wringing her hands and weeping most pitifully, accusing herself all the while of having killed poor little Mattie; having first exclaimed, "Oh, Doctor! you are too late; poor little Mattie is gone!" Indeed, to all appearances, it seemed to be true.

Upon inquiry I learned that the little girl had been seized, a little while after she had taken the powder, with a convulsion, which, however, soon passed off, leaving her in a lethargic condition, and that she had breathed more and more slowly until she had ceased to breathe altogether.

In answer to a question it was stated that she had breathed once only in the last several minutes.

The face was very pale; the lids partially closed; the pupils a little more dilated than normal; the muscles of the whole body completely relaxed; no perceptible respiration; no pulse, radial, temporal, carotid or cardiac, could be at all detected. Indeed, it looked as if the little patient had breathed her last.

Without hesitating a moment—believing I could do no harm—I dissolved or mixed about ten grains of the solid extract of nux vomica in three or four teaspoonfuls of home-made peach brandy, and proceeded at once to inject the whole of it beneath the skin of the little patient's arms and thighs. A few moments only were necessary for this procedure.

In about a minute after the last injection was made, the face became suddenly very red, and in an instant thereafter the little patient was seized with as severe a tetanic convulsion of emprosthotonus character.

This convulsion seemed to last several minutes, during which time the patient writhed fearfully, having her head drawn forward and downward. After convulsion subsided the little girl fell back upon the pillow and remained quite motionless—the whole system now completely relaxed—except that respiration and pulse-beat were nearly normal for several minutes, when she raised up and sat upright upon the bed and asked for water. After drinking freely she remained sitting up, and conversed intelligently, but in rather an excited and rapid manner, the remainder of the afternoon.

With the exception of a slight fever for a day or two, and several inconsiderable abscesses at points of puncture, the little patient passed on to a rapid and complete recovery.

Case 2.—Early on the morning of the twenty-sixth of May, 1882, I was summoned at two p. m. to see Mrs. Lawson, age fifty-eight. Upon inquiry I learned it was thought that she had taken too much opium.

I found patient in bed ; coma well established ; pupils very much contracted and immovable; muscles of arms and lower limbs very much relaxed; extremities cold; face puffy and pallid; pulse 128; temperature 102°; respiration 3, sometimes less; stertor prominent.

Patient could not be aroused by loud calling, nor by any attempt at the infliction of pain, nor by any other means. Husband could not tell how much opium his wife had taken; she was unaccustomed to its use so far as he knew. She had taken the opium to check diarrhœa.

The patient's condition resembled cerebral apolexy so closely that I was in much doubt with regard to a correct diagnosis. The evenly contracted, immovable pupils suggested opium poisoning, while the pulse and temperature indicated apoplexy. The fact that she had been taking pills of the drug at regular hours the day before, and several times during the night, and the gradual progress of stupor, seemed to confirm the toxical nature of her condition.

Acting upon this assumption, and remembering the case of the little girl, I commenced treatment by injecting subcutaneously thirty drops of the fluid extract nux vomica (P., D. & Co.), and awaited results.

There having been no perceptible change in a half hour, 30 drops more were injected into the opposite arm. This operation was repeated twice again at intervals of half an hour each, the only result being an increase in respiration, which was now six per minute. After awaiting another half hour I repeated the operation, injecting the same quantity of medicine as before. This resulted in slight convulsive movements, extending throughout the entire system. The legs were drawn up, and patient gave utterance to a deep groan.

Two hours had now passed, and the patient had received into her body 150 drops of the nux vomica with but very little improvement.

I now waited one hour and found the following condition: Pulse, 126; temperature, 102 2-5; respiration, 8.

Coma had given place to stupor; the patient could be partially aroused; would look up and answer questions, but in monosyllables, incoherently and very drowsily, and would instantly return to a state of unconsciousness, when snoring was again deep and hoarse.

I decided to test the matter at once so far as the nux vomica was concerned, so I charged the cylinder of my syringe with a little more than a drachm (all it would hold) of the medicine, and injected it near the xiphoid cartilage beneath the integument. The effect was magical. The patient was thrown into a severe epileptiform convulsion which lasted some moments. This passed off, but was soon followed by another, and still another, until she had had six very severe ones. The seventh was mild and no more followed.

It was now nearly 6 p. m.; nearly four hours had passed since I had first seen patient. I had used nearly three and a half drachms of the nux vomica in this space of time, hypodermically, with the following result: Pulse, 128; temperature, 103; respiration, 18. Pupils a little more contracted than normal. Patient would talk intelligently, and no drowsiness nor disposition to sleep.

I called early the next morning. Found pulse, 122; temperature, 100½; respiration, 18. Patient had slept but little during the night. Had had no more convulsions. Indeed, had entirely recovered from the effects of the opium poisoning, and lived on a little more than a year.

It may be well here to remark that Mrs. Lawson was suffering from a constant diarrhœa dependent upon hepatic cirrhosis or interstitial hepatitis, and previously to the diarrhœa she had suffered from severe dyspnœa dependent upon ascites extensively developed. Œdema of the lower extremities had also progressed to the point of rupture of the epidermis just above and about the ankles. These dropsical symptoms had, however, nearly entirely subsided at the time of the extreme opium narcosis. These facts are mentioned because a morbific process so extensive may have exerted some influence modifying the effect of the poison or its antidote.

With regard to the first case, it is at least possible that the pricking of the flesh, the wounding of the delicate fibres of the peripheral nerves by the introduction of hypodermic needle may have produced a shock sufficient to have excited momentary reaction in the whole nervous system, and contributed somewhat to the ultimate result.

Again, the small amount of brandy used, probably half an ounce, in which was dissolved the nux vomica, may have exerted some energy, and to this agent also may be ascribed at least some of the wonderful result. But would either, or both together, have exerted so prompt and lasting an antidotal influence as was here seen? Is it not evident that these alone could lend but feeble and transient antagonism to so large a portion of the powerful drug, holding so complete sway in the system of so feeble a subject? Would any other drug or drugs, or any other means whatsoever, have acted so promptly, completely and permanently as did the nux vomica in this case?

Opium, in poisonous quantities, is sedative, narcotic, anæsthetic and myo-paralytic; produces giddiness, stupor, insensibility and paralysis.

The appearance of the whole body and the expression of the countenance is that of complete relaxation; painless, deep and perfect repose.

Nux vomica, on the contrary, is a powerful excitant of the cerebro-spinal system; and in poisonous portions produces great agitation and anxiety: first, epileptiform and finally tetanic convulsions.

Instead of the complete relaxation of the whole voluntary muscular system; the painless, deep and perfect repose so characteristic of extreme opium narcosis, we witness "great agitation and anxiety "(1) ; "the most violent contortions of all the voluntary muscles and expressions of most intense agony "(2); "no poison causes so much torture"(3).

Now, if pain is the natural physiological antagonist of ease; agitation, restlessness, that of repose ; immobility that of relaxtion ; spasm that of paralysis; hyperæsthesia that of insensibility, why, I ask in the name of philosophical inquiry, why is not nux vomica the natural physiological antagonist of opium?

---

(1). Dr. Marshall Hall. (2). L. D. Ford, M.D., LL.D. (3). Isaac Hays, M. D.

Finally, to render this antagonism more complete—if it be possible so to do—it may be well to suggest, in this connection, the combination—in suitable proportions—with one of the salts of strychnin, some of those of caffein or thein, inasmuch as these last are said to possess the power of stimulating or exciting to a remarkable degree the cerebral functions, thus exalting cerebration, which is the natural counterpart of unconsciousness.

[It strikes us that the doses of nux vomica given in the cases reported above are hazardous. We think this drug should be administered cautiously.—EDITORS.]

